# Recent Advances in Underwater Signal Processing

**DOI:** 10.3390/s23135777

**Published:** 2023-06-21

**Authors:** Xuebo Zhang, Haixin Sun

**Affiliations:** 1 Whale Wave Technology Inc., Kunming 650200, China; xby_zhang@nwnu.edu.cn; 2 School of Informatics, Xiamen University, Xiamen 361005, China

The ocean, covering 71% of the Earth’s surface, is integral to human life. To solve its mysteries, equipment such as sonar and radar have emerged to perform topography, underwater communication, target detection, positioning, imaging, and ocean monitoring. Recent advances in signal processing and electronic technology have propelled new theories, mechanisms, and processing technologies for underwater equipment to a new stage.

This Special Issue aims to highlight recent advancements, developments, and applications in underwater signal processing methodologies, including characterization, simulation, real data processing, as well as applications to underwater engineering.

The editorial of this Special Issue introduces a total of 12 articles, which are divided into four types: (1) optimization of ship navigation, (2) underwater acoustic communication, (3) underwater acoustic signal recognition, and (4) underwater detection and positioning. The specific breakdown is as follows: two articles are introduced for the first type, four articles for the second type, three articles for the third type, and three articles for the fourth type.

## 1. Optimization of Ship Navigation

Ship navigation optimization has received much attention in recent years due to the increasing demand for ocean resources and space, presenting various challenges to ocean management and security. The need to improve ship navigation safety and detect ship operating trajectories is growing. In this issue, two articles present valuable methods and ideas.

In [1], researchers discuss the significance of atomic interference gravimeters for underwater navigation assistance. The instrument requires high precision to measure weak gravitational signals. To ensure accuracy, vibration isolation is essential in reducing external interference. The article reviews three vibration isolation methods: passive isolation, active isolation, and vibration compensation. It also highlights the direction of vibration compensation improvement as a future development trend.

In [2], researchers address the storage, management, analysis, and mining of ship target data. It designs and develops the overall structure and functional modules of the Ship Trajectory Data Management and Analysis System (STDMAS), proposing a ship identification method based on motion characteristics. The system is user-friendly, easy to maintain, and expandable, meeting the actual needs of ocean target data management, analysis, and mining. However, the current processing capacity for AIS data is limited. Future research can utilize big data algorithms and cloud computing architecture to improve the efficiency of processing massive data.

## 2. Underwater Acoustic Communication

Underwater acoustic communication utilizes sound waves to propagate through water and has various applications, including ocean detection, underwater sensing, and underwater operations. In recent years, underwater acoustic communication technology has gained increasing attention and research. In this article, we introduce four new methods to enhance the effectiveness of waterborne acoustic communication from different perspectives.

The article [3] explores the impact of temporal and spatial fluctuations of the ocean acoustic field on waterborne acoustic communication. Researchers have theoretically derived the fluctuation of signal intensity concerning changes in horizontal distance, signal frequency, bandwidth, and deployment depth, which were further verified through simulation and analysis experiments in the Yellow Sea acoustic field. The experimental results showed that a vertical array should be used for reception in shallow-water acoustic communication to improve the signal-to-noise ratio and system reliability. In addition to the acoustic field, corresponding achievements have been made in underwater signal sampling and transmission. Ref. [4] proposes a signal transmission and reception method for sonobuoys based on an autoencoder. By using an autoencoder at the transmission and reception ends, signal compression and restoration can be efficiently achieved, reducing the impact of environmental noise and improving the reliability of signal transmission. Furthermore, the article [5] designs a hardware/software platform for measuring channels and testing transmission technology under actual conditions in the ultrasonic band, allowing for the analysis and design of new underwater communication system solutions.

The overview article [6] takes the routing protocol as a starting point and discusses its various aspects, such as the concept and causes of void regions and the main challenges researchers face when designing routing protocols. The most advanced void avoidance protocol using OR technology is studied in-depth. We believe that these research results will provide robust support for applying and promoting waterborne acoustic communication technology.

## 3. Underwater Acoustic Signal Recognition

We have achieved significant progress not only in underwater acoustic communication but also in underwater acoustic signal recognition. This has become a prominent research field, with applications in underwater communication, ocean exploration, sonar detection, and other areas.

In [7], researchers proposed an EVMD algorithm that overcomes the accuracy limitations of VMD execution in the field of underwater acoustic communication. Simulation and experimental results show that this method has a recognition rate superior to traditional ship-radiated noise feature extraction methods, with a recognition rate of up to 96.6667%. However, the paper only discusses VMD mode numbers, and future work will consider optimizing the number of models and quadratic penalty terms for achieving higher decomposition accuracy.

Reducing noise and efficiently acquiring target underwater acoustic signals by sensors are equally crucial in signal recognition. In [8], the researchers proposed a method using Hidden Markov Models (HMM) to detect sequence acoustic data without separate training data. The stability and accuracy of detecting signals of interest (SOI) were improved using genetic algorithms and multiple measurements. Therefore, the multi-measurement GA-HMM exhibited excellent performance in both passive and active acoustic data.

Similarly, we have made significant progress in image transmission recognition. In [9], a normalization-based adaptive modulator (INAM) was proposed, which amplifies pixel deviations through adaptive predictive modulation factors. INAM was introduced into the learning of image-adaptive 3D LUT for underwater image enhancement, achieving good results.

## 4. Underwater Detection and Positioning

In addition to the previously mentioned fields, the importance of underwater detection and positioning technology in ocean science cannot be understated. Nowadays, this technology has been extensively utilized in ocean exploration, ocean resource development, underwater safety monitoring, and other areas. However, the complex marine environment can significantly impact the accuracy of underwater detection, and developing this technology entails overcoming substantial challenges. This issue of the *Sensors* journal presents three articles on underwater detection and positioning that are of significant importance for advancing this technology.

To address the issue of low signal-to-noise ratios in the underwater environment, which poses challenges for active sonar in detecting, tracking, and identifying underwater targets, the article [10] proposes a Tacotron model-based deep neural network (DNN) approach for active sonar signal synthesis. This method applies the Tacotron model to sonar signal synthesis and successfully synthesizes data that are almost identical to the data used for training, as confirmed by spectral comparison, attention result inspection, and MOS testing.

To improve the tracking performance of low SRR underwater targets, the article [11] proposes a particle filtering track-before-detect algorithm based on the knowledge-aided (KA-PF-TBD) algorithm. This method maximizes the utilization of prior information on the underwater diver target and establishes a set of multi-directional motion models to address the mismatch between conventional model sets and actual target motion.

In [12], researchers conducted a full-scale experiment simulating the underwater localization of magnetic sensors. Two natural computing algorithms were utilized to solve the signals generated by the known ferromagnetic object trajectory, successfully determining the positions of eight magnetometers. The methods performed exceptionally well, particularly in the multi-target version, accurately determining the position of the sensor with a relative error of 1% to 3%. The near sensor and the far sensor exhibited absolute errors of between 20 and 35 cm, respectively. These three papers illustrate the continual development and innovation in underwater detection and positioning technology.

## 5. Conclusions

The theme of this Special Issue focuses on underwater signal and ocean signal processing. This Special Issue highlights 12 articles that can be divided into four categories: optimization of ship navigation [1,2], underwater acoustic communication [3,4,5,6], underwater acoustic signal recognition [7,8,9], and underwater detection and positioning [10,11,12]. In addition to traditional underwater acoustic signals, research objects also include underwater sensors, underwater environments, ships, underwater images, etc. Therefore, in the field of underwater acoustic communication, in addition to traditional signal processing and analysis methods, there are many related technologies and applications worthy of research. In addition, in terms of algorithm design, the development of artificial intelligence algorithms also provides new solutions to analyze and process underwater signals. Signal processing algorithms that combine artificial intelligence algorithms with underwater signal processing technology will be a very important development trend in the future.
